# COVID-19 Infection, Vaccination, and Antibody Levels: Investigating Correlations through a Cohort Study

**DOI:** 10.3390/vaccines11071258

**Published:** 2023-07-19

**Authors:** Gözde Akkuş Kayalı, Seyfi Durmaz, İrem Nur Şahin, Betül Akkul, Raika Durusoy, Funda Karbek Akarca, Sezgin Ulukaya, Candan Çiçek

**Affiliations:** 1 Department of Medical Microbiology, Faculty of Medicine, Ege University, Izmir 35100, Turkey; irem_nur_sahin@hotmail.com (İ.N.Ş.); akkulbetul13@gmail.com (B.A.); candan.cicek@ege.edu.tr (C.Ç.); 2 Department of Public Health, Faculty of Medicine, Ege University, Izmir 35100, Turkey; seyfidurmaz@gmail.com (S.D.); raika.durusoy@ege.edu.tr (R.D.); 3 Department of Emergency Medicine, Faculty of Medicine, Ege University, Izmir 35100, Turkey; funda.karbek.akarca@ege.edu.tr; 4 Department of Anesthesiology and Reanimation, Faculty of Medicine, Ege University, Izmir 35100, Turkey

**Keywords:** SARS-CoV-2, anti-nucleocapsid, anti-spike, healthcare worker

## Abstract

Aim: The objective of this study was to explore the potential correlation between COVID-19 infection or vaccination and levels of anti-nucleocapsid (anti-N) and anti-spike (anti-S) antibodies. Methods: Among 6050 healthcare workers at the Ege University Hospital, a cohort study with 162 participants divided into three arms with 54 participants each was conducted. The three groups were selected as follows: those diagnosed with COVID-19 and not vaccinated (group 1), those diagnosed with COVID-19 and subsequently vaccinated with CoronaVac (group 2), and those not diagnosed with COVID-19 but vaccinated with two doses of CoronaVac (group 3). Antibody levels measured at the sixth month of follow-up were defined as the primary outcome. Results: At the sixth month, all serum samples tested positive for anti-S. Anti-S levels were found to be significantly higher in group 2 than in the other groups (*p* < 0.001). There were no differences in antibody levels between groups 1 and 3 (*p* = 0.080). Average antibody levels were found to be lower in office workers and males. Anti-N antibodies were found to be positive in 85.1% of subjects at the sixth month. In group 2, anti-N antibodies were detected in all samples at the sixth month. Anti-N antibody levels were not significantly different between groups 1 and 2 (*p* = 0.165). Groups 1 and 2 had significantly higher antibody levels than group 3 (*p* < 0.001). Conclusions: Vaccination or infection provide protection for at least 6 months. Those who have previously been diagnosed with COVID-19 do not need to be vaccinated in the early period before their antibody levels decrease.

## 1. Introduction

In Wuhan, in the Hubei Province, China, a significant increase in pneumonia cases has been recorded since December 2019, and genetic analysis has revealed that the microbial agent responsible was a new type of coronavirus (2019-nCoV or SARS-CoV-2). Subsequently, the disease has quickly spread throughout China and the rest of the world, causing a pandemic. Vaccine trials have been accelerated due to the rapidly increasing number of patients and the deleterious effects of the COVID-19 pandemic. Following Phase 3 clinical trials, safe and effective vaccines have been approved for immediate use during the pandemic. The first approval for emergency use was granted by the WHO on 31 December 2020, for an mRNA-based vaccine from BioNTech (BNT162b2) (Pfizer, Mainz, Germany) [1]. The CoronaVac vaccine (Sinovac Life Science Co., Ltd., Beijing, China) was validated for emergency use by the WHO on 1 June 2021 [2]. Healthcare workers have been identified by the WHO and the Turkish Ministry of Health as one of the high-priority groups for mass vaccination [3]. The whole-virus inactivated vaccine CoronaVac was the first vaccine to be approved for use by healthcare workers in Turkey on 13 January 2021. The first mass vaccination started to be administered on 14 January 2021, and the second doses were administered >4 weeks later. BioNTech vaccines were introduced in Turkey on 12 April 2021 [4] and made available to previously CoronaVac-vaccinated healthcare workers on 1 July 2021.

Although antibodies found after natural infection were predominantly anti-N, most vaccines stimulate the production of antibodies against the spike region [5]. These antibodies have influenced diagnostic tests [6,7]. Furthermore, because spike proteins bind to target cells through their receptor binding domain (RBD), they are the target of neutralizing antibodies [8]. In addition to PCR tests, serological assays can be used to make a diagnosis. It is controversial whether these tests can be used to predict re-infection in high-risk contact situations. Further research is needed to better understand the significance of serological tests [9].

During the early stages of the COVID-19 pandemic, it was important to monitor antibody responses in healthcare workers. Compared with other occupations, healthcare workers had a higher risk of COVID-19 exposure at the beginning of the pandemic. To understand the effects of SARS CoV-2 exposure, we aimed to examine antibody levels in healthcare workers and check for increased antibody responses to repeated SARS CoV-2 micro-contacts in healthcare workers providing healthcare to people diagnosed with COVID-19.

The objective of this study was to explore the potential correlation between COVID-19 infection or vaccination and the levels of anti-nucleocapsid (anti-N) and anti-spike (anti-S) antibodies.

## 2. Materials and Methods

Between November 2020 and August 2021, a cohort study was conducted on selected individuals from a total of 6050 healthcare workers (HCWs) at the Ege University Hospital, Turkey. The study groups were selected from 860 HCWs who already had COVID-19 (605 vaccinated and 255 not vaccinated) between December 2020 and January 2021 and from 4700 HCWs who received the CoronaVac vaccine between January and February 2021.

The sample size was determined to be at least 135 for a three-group study with a medium effect, a 5% margin of error, a 95% confidence level, and 80% power. A reserve of 20% has been added to cover a possible loss in follow-up under pandemic conditions. Among the 162 participants included in the study, 54 were randomly assigned to the three follow-up groups:

- Group 1: Participants were diagnosed with COVID-19 and not vaccinated.
- Group 2: Participants were diagnosed with COVID-19 and subsequently vaccinated with CoronaVac.
- Group 3: Participants who had been vaccinated with two doses of CoronaVac in the Turkish Ministry of Health vaccination program (without prior diagnosis of COVID-19)

**Figure 1:**
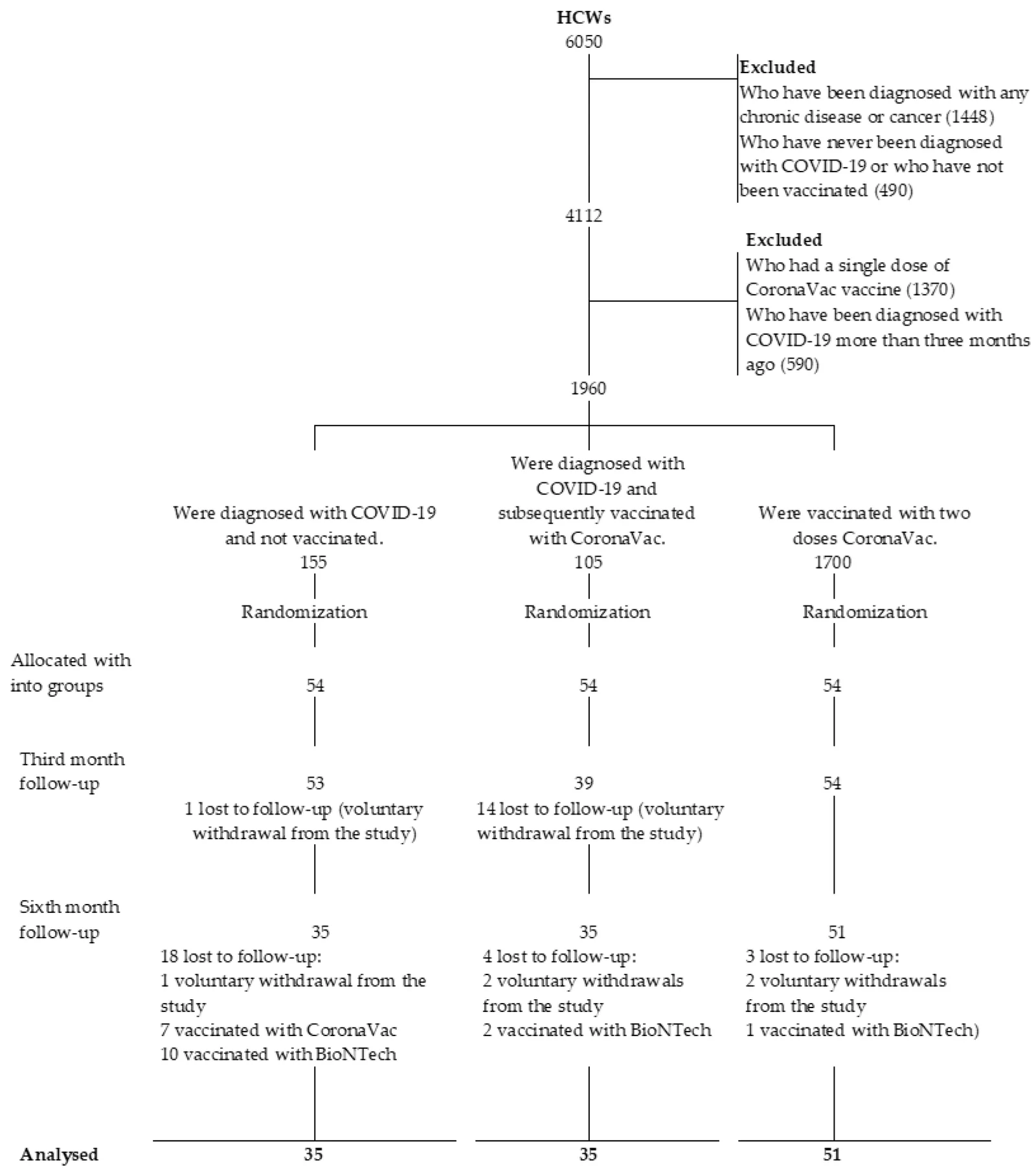
Flowchart of participants in the cohort study.

Randomization was conducted using a computer-generated random list. The study started with 146 randomly selected individuals, and some data were lost for various reasons during the 6-month follow-up period (Figure 1).

### 2.1. Data Collection

The study groups were regularly monitored by the hospital‘s Occupational Health and Safety Unit (OHSU). SARS-CoV-2 was also tested in participants who had COVID-19 symptoms or had been in contact with a person known to be infected. The recorded follow-up data of all participating healthcare workers was obtained from the OHSU.

A data collection form that included 28 questions was used to collect data on the characteristics of the participants, such as socio-demographic characteristics, co-morbidities, occupation, unit of employment, degree of exposure to COVID-19 in this unit, vaccination status, disease process, and clinical status of those infected (mild, moderate, and severe). The data collection form was piloted and revised before being used by participants.

The participants were informed of the study, and written consent was obtained. Blood samples were collected from groups 1 and 2 at three and six months after the first day of COVID-19 symptom onset and/or positive PCR test results, respectively. Blood samples from group 3 were collected at three and six months following the administration of the first dose of CoronaVac. Nasopharyngeal swab samples (NPs) were also collected from all participants at the time of blood collection.

### 2.2. Laboratory Assay

Serum samples were tested for two different types of antibodies (Elecsys^®^ Anti-Spike IgG antibody (Anti-S) and Elecsys^®^ Anti-Nucleocapsid IgG/IgM antibody (Anti-N), Roche Diagnostics, Mannheim, Germany) on the Cobas e 411 analyzer. The quantitation range for the Anti-S test was 0.8–250 U/mL. U/mL values were used as binding antibody units (BAU/mL) according to the WHO [10,11,12]. Values of ≥0.8 U/mL were considered positive according to the manufacturer’s recommendations. The results of the anti-N tests were determined according to a cut-off index (COI; signal sample/cutoff). The index values of COI ≥1.0 were considered positive according to the recommendation of the manufacturer.

SARS-CoV-2 RNA was investigated by the reverse transcriptase-polymerase chain reaction (RT-PCR) method (Bio-Speedy COVID-19 qPCR, Bioexen, Istanbul, Turkey).

### 2.3. Measures and Outcomes

Variables were determined as age, gender, occupation, unit of employment, degree of exposure to COVID-19 in that unit, vaccination status, and diagnosis of SARS-CoV-2 infection. Measurements of antibody levels in blood samples collected atthree and six months were used as outcomes.

Antibody levels measured at the sixth month were defined as the primary outcome, and antibody levels measured at the third month were evaluated as the secondary outcome. In the early phase of the pandemic, antibody measurements at the sixth month have been considered a critical period both to demonstrate a sufficient amount of antibody switch and to minimize the impact of other confounding factors. As process outcomes, antibody levels measured at the third month were compared with antibody levels measured at the sixth month.

### 2.4. Statistics

The Statistical Package for Social Sciences (SPSS) version 25.0 (IBM Inc., Armonk, NY, USA) was used to analyze the data. The chi-square, Student’s *t*-test (or Mann-Whitney U), and ANOVA (or Kruskal-Wallis) tests were used to compare the differences between groups, the presence or level of antibodies, and group characteristics. Regression analysis was used to determine the impact of being in different study groups on the presence of antibodies. Pearson’s Correlation and Linear Regression analyses were performed to examine the correlation between time and antibody responses. The level of significance was set at *p* < 0.05.

## 3. Results

One hundred and sixty-two individuals who met the inclusion criteria of the three groups contributed to the study, among whom 14 participants who had been diagnosed with COVID-19 or had been vaccinated withdrew from the study without their samples being collected. Twenty participants who changed their vaccination schedule were excluded from the study. In addition, five participants voluntarily withdrew from the study. Among all participants, only one had a positive SARS-CoV-2 RNA PCR test result during the study sampling, and this participant was therefore excluded five months after the first dose of vaccination. Analyses were continued for the remaining 121 individuals (Figure 1).

The mean age of the participants was 39.1 ± 10.7, and 71.2% (*n* = 104) of the participants were female. The distribution of factors that might influence antibody formation within the groups, their descriptive characteristics, and the clinical status of individuals with COVID-19 are shown in Table 1. 

### 3.1. Levels of Anti-S

**Figure 2:**
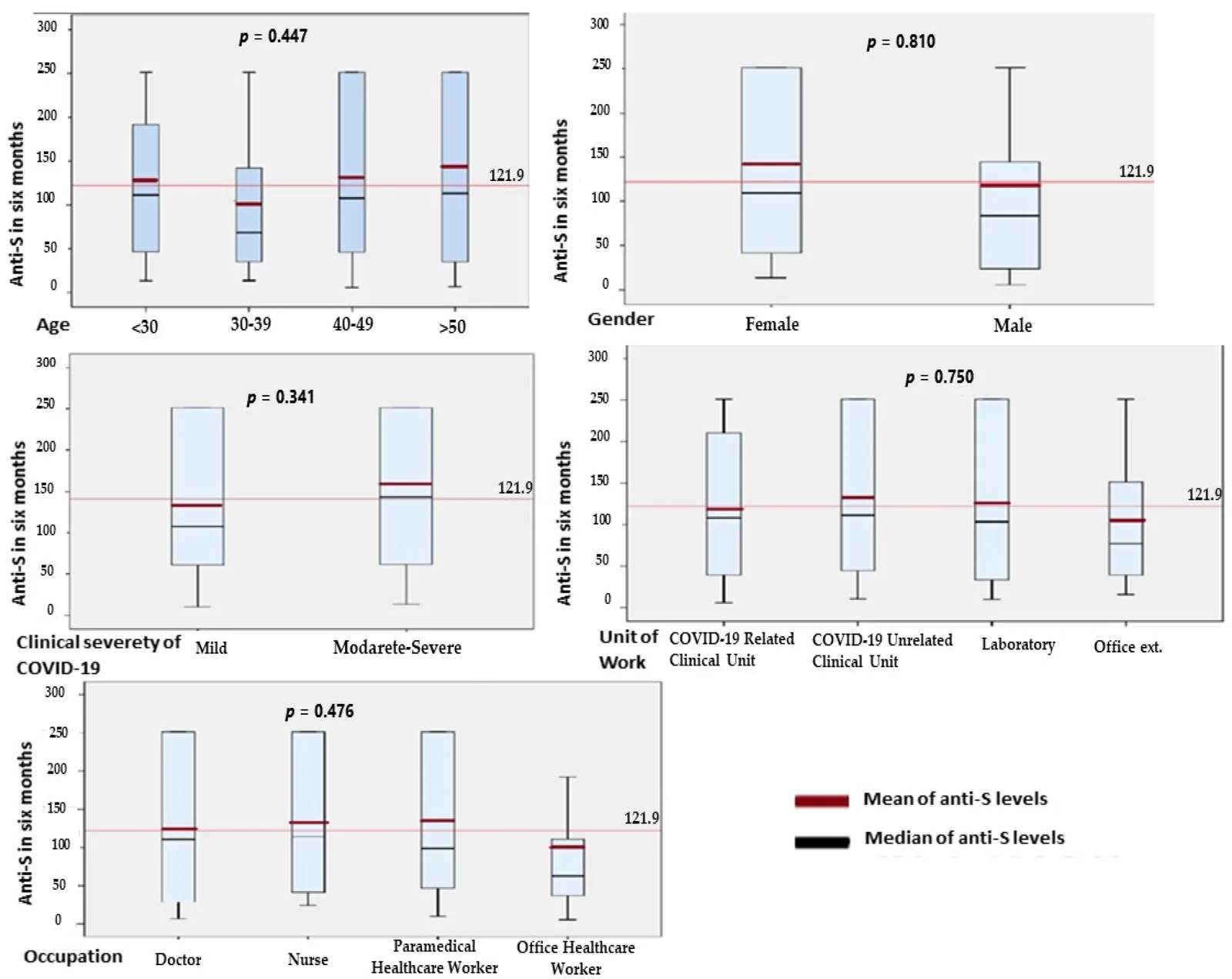
Effects of variables on antibody levels Anti-S: Anti-Spike IgG; Anti-N: Anti-Nucleocapsid IgG/IgM; COVID-19: Coronavirus Disease 2019. The long red line in all figures crosses through the point of 121.91 U/mL, representing the mean antibody level of Anti-S for all participants.

The anti-S test was positive for all serum samples from participants at 6 months (Table 2). The anti-S level in group 2 was found to be significantly higher than that in the other groups by linear regression analysis at sixth month measurements (*p* < 0.05). There were no differences in antibody levels between groups 1 and 3 (*p* = 0.080) (Table 3). The mean antibody levels were lower in office workers and male participants (Figure 2). In addition, there were no differences in the antibody levels of participants by age (*p* = 0.447), gender (*p* = 0.81), occupation (*p* = 0.475), or unit of employment (*p* = 0.75) (Figure 2). In addition, the results of the analysis at 3 months were similar to those at 6 months for the same variables.

### 3.2. Levels of Anti-N:

The anti-N was detected in 85.1% of participants at 6 months. In group 2, the anti-N was found in all participants’ analyses at the sixth month (Table 2).

There was no significant difference between the anti-N levels in groups 1 and 2 (*p* = 0.165) at six months. The antibody levels in groups 1 and 2 were significantly higher than those in group 3 (*p* < 0.001) (Table 3). There were no differences in the participants’ antibody levels in terms of age (*p* = 0.776), gender (*p* = 0.157), occupation (*p* = 0.273), or unit of work (*p* = 0.193). In addition, the results of the analysis at the third month were similar to those at the sixth month for the same variables.

### 3.3. Comparison of Antibody Levels at Three and Six Months, Clinical Status, and One Participant Who Developed COVID-19 during Follow-Up

**Figure 3:**
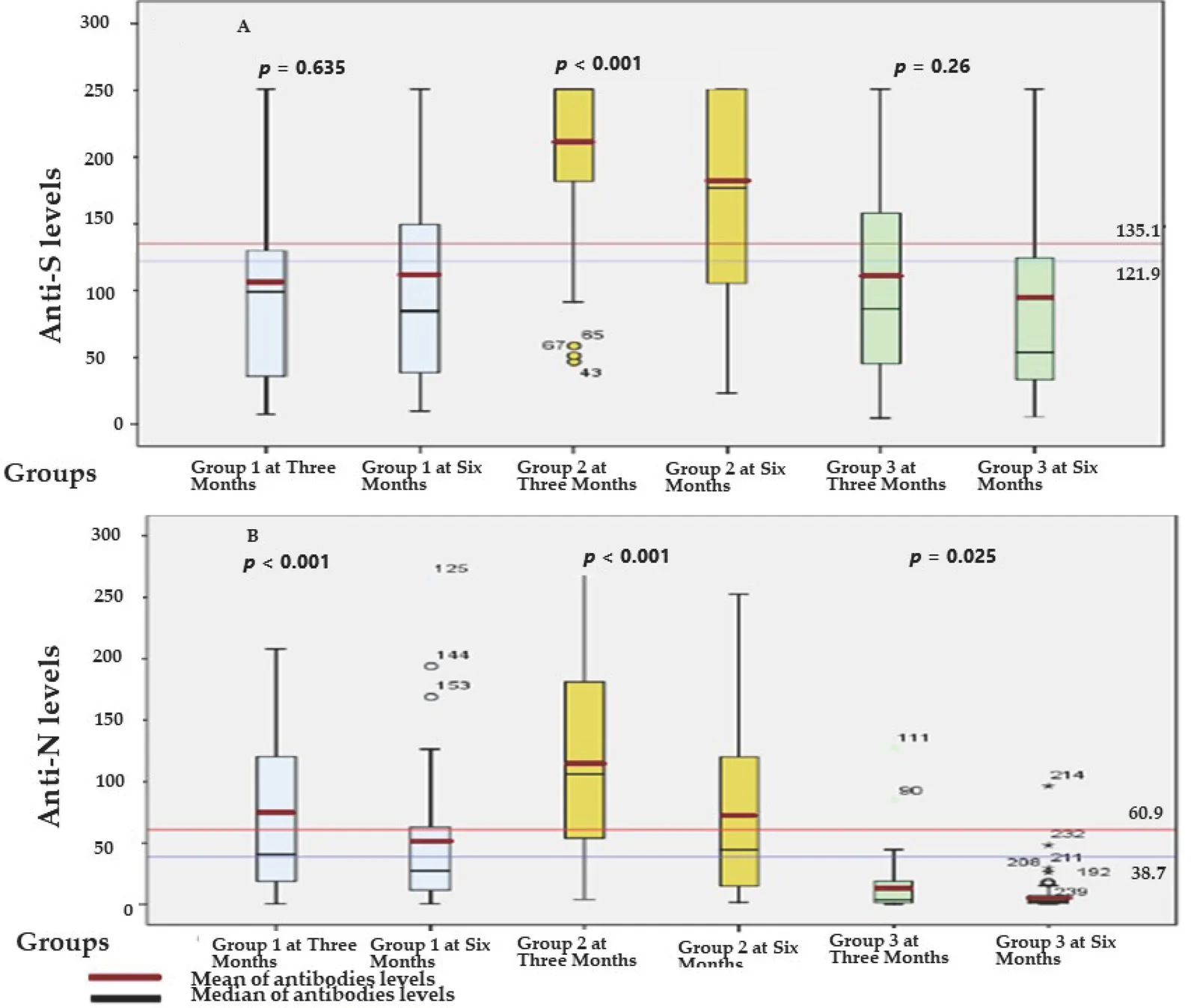
(**A**) Anti-S levels compared at the third and sixth months. The long red line in this figure crosses through the point of 135.1 U/mL, representing the mean antibody level of the anti-S for all participants in the third month. In addition, the long blue line in this figure crosses through the point of 121.91 U/mL, representing the mean anti-S antibody level for all participants at 6 months. (**B**) Anti-N Ab levels compared at the third and sixth months. The long red line in this figure crosses through the point 60.93 COI, representing the mean antibody level of the anti-N for all participants in the third month. In addition, the long blue line in this figure crosses through point 38.68 COI, representing the mean antibody level of the anti-N for all participants at the sixth month.

The measured levels of anti-S at the third and sixth months were similar in groups 1 (*p* = 0.635) and 3 (*p* = 0.26). In group 2, anti-S antibody levels at the sixth month were significantly lower than antibody levels at the third month (*p* < 0.001). In group 2, values decreased significantly by 35.9 RAU/mL from the third to the sixth month (*p* > 0.001). An increase in antibody of 3.5 RAU/mL was observed in group 1 (*p* > 0.05). The median duration of post-illness vaccination for COVID-19 participants in group 2 was 33 (Q1 = 29–Q3 = 40) days. In group 2, participants with a longer time between COVID-19 and vaccination with CoronaVac had higher anti-S levels measured at 6 months (r(33) = 0.433, *p* = 0.009), and these two variables showed a linear relationship (anti-S level (IU/mL) = 100.38 + time(day) × 1.845). Mean anti-S levels corresponding to quartile intervals of vaccination days after COVID-19 were as follows: −214.8 U/mL (SD = 76.5) in people vaccinated >40 days later; 182.3 U/mL (SD = 83.2) in people vaccinated 33–40 days later; 144.5 U/mL (SD = 79.8) in people vaccinated 29–33 days later; and 146.4 U/mL (SD = 79.3) in people vaccinated <29 days later (*p* = 0.182). In the same participants, as the time between COVID-19 and vaccination increased, anti-N levels also increased, and a linear relationship was found between them (r(33) = 0.436, *p* = 0.009), (anti-N level (COI) = 10.346 + time(day) × 1.573). The mean anti-N levels corresponding to the quartile intervals of vaccination days after COVID-19 were as follows: −110.2 COI (SD = 87.6) in people vaccinated after 40 days, 61.2 COI (SD = 67.9) in people vaccinated after 33–40 days, 65.5 COI (SD = 61.1) in people vaccinated after 29–33 days, and 48.6 COI (SD = 46.7) in people vaccinated <29 days (*p* = 0.579). Anti-N levels were lower at the sixth month compared to the third month in all groups (*p* > 0.05) (Figure 3).

There was a positive correlation between anti-N and anti-S levels at the third month (r(119) = 0.656, *p* < 0.001). Furthermore, between anti-N and anti-S levels at the sixth month (r(119) = 0.533, *p* < 0.001).

- Group 1: Participants who had been diagnosed with COVID-19 and who had not yet been vaccinated.
- Group 2: Participants who had been diagnosed with COVID-19 and who were subsequently vaccinated with CoronaVac.
- Group 3: Participants who have been vaccinated with two doses of CoronaVac in the Turkish Ministry of Health’s vaccination program (without prior diagnosis of COVID-19)

The clinical status of people with COVID-19 had no effect on the antibody levels that were measured at the third and sixth months (*p* = 0.341) (Figure 2).

During follow-up, one patient in group 3 developed COVID-19 symptoms at five months, and the PCR test result was positive. This participant’s Anti-S and Anti-N antibody levels were 57.8–25.9 U/mL and 2.29–3.90 COI at three months and five months, respectively. Mild symptoms were observed throughout the course of the participant’s disease.

## 4. Discussion

Six months of blood samples were collected from participants diagnosed with COVID-19 and/or vaccinated with CoronaVac. Higher levels of anti-S and anti-N antibodies were detected in the group vaccinated after COVID-19 than in the other groups, and this difference persisted for six months.

Seventy-two percent of the study participants and 65% of the healthcare workers at the Ege University Medical Hospital were female. The gender distribution in the main cluster and in the study group was similar, and the distribution in the groups was homogeneous. Although females had higher levels of anti-S antibodies in this study, no significant difference was found between males and females. Studies have shown that antibody responses are generally higher in the female gender [13,14,15].

When the distribution by occupational group was examined, no homogenization between the groups was achieved, as the diagnosis of COVID-19 was lower among doctors and office workers. Although the mean anti-S level was lower in office laboratory workers, no statistical difference was observed in further analyses.

All study participants were positive for anti-S antibodies and remained positive for 6 months. At the sixth month of follow-up, the mean anti-S level in group 2 was higher than the mean antibody level in group 1, and vaccination in the first three months of the disease significantly increased the antibody level. However, when we examined the antibody changes within the groups, the mean antibody level of group 2 decreased significantly by 35.9 RAU/mL from the third to the sixth month. In addition, an increase in antibody of 3.5 RAU/mL was observed in group 1 [16]. This increase did not make a significant difference in this group in the third and sixth months. When we evaluated this result, the increase continued during the first three months in people who had COVID-19 and had never been vaccinated, and it was found to be higher at the 6-month measurement than at the 3-month measurement. Previous studies have reported that the peak time of anti-S antibodies is in the range of 126–229 days [17]. Since the measurement period in this study was six months, it was predicted that the peak antibody time of group 1 could be 180 days or longer.

The participants in group 2 were vaccinated within the first three months after infection. The first measurements in this group showed that the antibody level was higher than that in the other groups, and this difference lasted for six months [15,18,19]. However, in this group, in contrast to group 1, antibody levels were found to be lower at month six than at month three. In group 2, participants with more time between COVID-19 diagnosis and vaccination had higher antibody levels at the sixth month. This could be because early vaccination interferes with the normal course of innate immunity. Based on the findings of this study and the intervals at which peak antibody levels have been reported in the literature, it is recommended that people with COVID-19 be vaccinated after the time of peak antibody production. In our study, the highest anti-S antibody levels were measured on day 180 in participants with natural immunity. Therefore, we recommend that people diagnosed with COVID-19 be vaccinated after an average of 180 days.

When the anti-S antibodies of groups 1 and 3 were examined, no difference was found between them in terms of the mean antibody levels at the third and sixth months. In terms of the antibody response, the importance of vaccination with two doses of CoronaVac over natural immunization is evident. Mean antibody levels similar to those of innate immunity were observed after vaccination with two doses of CoronaVac [19,20]. At this point, antibody studies should be supported by neutralization studies [9].

There are studies that have found a correlation between anti-S antibodies and neutralizing antibodies [16,21,22]. A threshold value of 15 U/mL for the detection of neutralizing antibody inhibitory effects has been defined by the manufacturer [23]. For the Elecsys Anti-SARS-CoV-2 S kit, 133 BAU/mL was determined in previous studies to predict the presence of neutralizing antibodies [24]. A protective neutralization level of 50% corresponds to a titration ratio of 1:10 to 1:30 in serum, which is estimated to be approximately 54 IU/mL (95% CI, 30–96 IU/mL) [25].

Notably, the assigned U/mL of Elecsys^®^ Anti-Spike IgG antibody is equivalent to Binding Antibody Units (BAU)/mL, as defined by the first World Health Organization (WHO) International Standard for Anti-SARS-CoV-2 immunoglobulin (NIBSC code 20/136). No unit conversion is required, and results reported in U/mL can be directly compared with other studies or results in BAU/mL [10,11,12]. In this context, all mean antibody values mentioned above were considered equivalent to BAU.

Anti-N antibodies occurred at a rate of 98.6% in individuals with a history of COVID-19, and this positivity rate decreased by only 4.3% at the six-month follow-up. Anti-N antibodies were observed in 80.4% of individuals vaccinated with CoronaVac without a diagnosis of COVID-19. During the six-month follow-up, the positivity rate in this group decreased by 7.9%. In the case of SARS-CoV-2 infection, the formation of anti-N antibodies is particularly important for diagnosis. When we compared the anti-N levels of groups 1 and 2, there was an increase of 21.9 COI in the group vaccinated after diagnosis, but no significant difference in antibody levels was found. In other words, when vaccinated in the first three months after COVID-19, there was no significant increase in anti-N antibody levels compared to no vaccination after diagnosis [20]. Furthermore, in group 2, anti-N levels at 6 months were lower in those vaccinated early after the disease.

The level of anti-N antibodies in participants diagnosed with COVID-19 was significantly higher than that in those participants who were vaccinated only. The mean COI level of 61.2 was measured in groups 1 and 2, six months after COVID-19 diagnosis. Furthermore, six months after immunization with the CoronaVac vaccine, which is an inactivated whole virus vaccine, a mean COI level of 7.8 was found, and 80% of the vaccine group participants were positive for the antibody [20]. This is an important point regarding the formation of anti-N antibodies after vaccination with CoronaVac as opposed to mRNA and vector vaccines. This confirmed the hypothesis that whole-virus vaccines induce an immune response against the antigenic structures of the nucleocapsid region [20]. However, this response is not as strong as the immunization that occurs after infection [20].

When changes in anti-N antibody levels were examined in the groups at 3 and 6 months, a significant decrease was seen [16]. This can be explained by the short half-life of anti-N antibodies [14].

When clinical conditions were examined according to WHO standards, the percentage of patients diagnosed with COVID-19 with a history of moderate to severe clinical disease was 31.9%. Antibody levels did not differ according to clinical status.

During follow-up, one participant in the vaccine-only group was found to be alpha (B.1.1.7) Sars-CoV-2 positive (UK variant). The participant was diagnosed in the fifth month after vaccination and had mild symptoms. No positivity was observed in any other group. Only 0.7% of participants had COVID-19.

Randomization could not be applied in group 1 because the number of workers diagnosed with COVID-19 but not vaccinated was low (*n* = 255). Participants in group 2 had variable vaccination times after diagnosis; therefore, the time of blood collection after the second dose of vaccination in this group varied between 8 and 44 days. There was only one person with a worse clinical status. Probably due to the limited sample size and the healthy worker/age-group effect, there were no severe cases enrolled in this study. As the BioNTech vaccine became available to healthcare workers in July 2021, it was impossible to extend the follow-up period. In this study, a six-month follow-up was conducted, and the number of participants was small. Studies with larger numbers of participants and longer follow-up periods are needed to investigate the prevention of re-infection. Another limitation was that this study did not investigate the presence of memory B and T cells, which might also contribute to immune persistence. Moreover, neutralizing antibodies have also not been investigated. The correlation between antibody levels and neutralizing antibodies and the significance of this correlation are unknown.

## 5. Conclusions

From this study, we can conclude that immunization after vaccination and infection provides protection for at least six months. However, the immune response resulting from an encounter with the SARS-CoV-2 antigen varies from person to person. Those who have previously been diagnosed with COVID-19 should not be vaccinated in the early period before the antibody level decreases. The antibody response is more strongly stimulated by innate immunity, or, in other words, by being diagnosed with COVID-19. This will be better understood in future studies on antibody responses to COVID-19 and vaccines. Further studies are needed.

## Figures and Tables

**Table 1:** Characteristics of the study groups (*n* = 146/121).

		Total Group (*n*; %)		Group 1 (53/35; 36.3/28.9%)		Group 2 (39/35; 26.7/28.9%)		Group 3 (54/51; 37.0/42.1%)		pA/pB
		nA/nB	%A/%B	nA/nB	%A/%B	nA/nB	%A/%B	nA/nB	%A/%B	
Gender	Female	104/89	71.2/73.6	34/23	64.2/65.7	31/29	79.5/82.9	39/37	72.2/72.5	0.270/0.261
	Male	42/32	28.8/26.4	19/12	35.8/34.3	8/6	20.5/17.1	15/14	27.8/27.5	
Age (39.1 ± 10.7)	<30	35/32	24/26.4	12/10	22.6/28.6	11/10	28.2/28.6	12/12	22.2/23.5	0.444/0.132
	30–39	44/37	30.1/30.6	18/12	34.0/34.3	11/10	28.2/28.6	15/15	27.8/29.4	
	40–49	36/28	24.7/23.1	12/8	22.6/22.9	13/12	33.3/34.3	11/8	20.4/15.7	
	≥50 *	31/24	21.2/19.8	11/5	20.8/14.3	4/3	10.3/8.6	16/16	29.7/31.4	
Occupation	Doctor	34/26	23.3/21.5	9/3	17.0/8.6	4/4	10.3/11.4	21/19	38.9/37.3	0.009/0.003
	Nurse	49/42	33.6/34.7	20/16	37.7/45.7	19/17	48.7/48.6	10/9	18.5/17.6	
	Paramedical HCWs	33/27	22.6/22.3	11/6	20.8/17.1	10/9	25.6/25.7	12/12	22.2/23.5	
	Office HCWs	30/26	20.5/21.5	13/10	24.5/28.6	6/5	15.4/14.3	11/11	20.4/21.6	
Unit of employment	COVID-19-related clinical unit	43/32	29.5/26.4	16/8	30.2/22.9	11/9	28.2/25.7	16/15	29.6/29.4	0.014/0.005
	COVID-19-unrelated clinical unit	57/48	39.0/39.7	21/14	39.6/40.0	19/18	48.7/51.4	17/16	31.5/31.4	
	Lab	19/18	13.0/14.9	2/1	3.8/2.9	3/3	7.7/8.6	14/14	25.9/27.5	
	Office	27/23	18.5/19.0	14/12	26.4/34.3	6/5	15.4/14.3	7/6	13.0/11.8	
Clinical severity of COVID-19	Mild	62/46	42.5/38.0	38/24	26.0/19.8	24/22	16.5/18.2	-	0/0	<0.001/<0.001
	Moderate	28/23	19.2/19.0	14/10	9.6/8.3	14/13	9.6/10.7	-	0/0	
	Severe	1/1	0.7/0.8	1/1	0.7/0.8	0/0	0/0	-	0/0	

* Only four participants were over 60 years of age and classified as “≥50 years”. nA (146), %A, pA: Distribution of study participants at baseline. nB (121), %B, pB: Participants included in the final analysis. Group 1: Participants who had been diagnosed with COVID-19 and who had not yet been vaccinated. Group 2: Participants who were diagnosed with COVID-19 and subsequently vaccinated with CoronaVac. Group 3: Participants who had been vaccinated with two doses of CoronaVac in the Turkish Ministry of Health vaccination program (without prior diagnosis of COVID-19).

**Table 2:** Distribution of antibody detection rates and levels by groups.

			Total Group		Group 1		Group 2		Group 3	
Antibody Detection Rate			(*n*; %)		(35; 28.9%)		(35; 28.9%)		(51; 42.1%)	
			*n*	%	*n*	%	*n*	%	*n*	%
sixth month	Anti-S	Positive	121	100	35	100	35	100	51	100
		Negative	0	0	0	0	0	0	0	0
	Anti-N (*p* = 0.002)	Positive	103	85.1	31	88.6	35	100	37	72.5
		Negative	18	14.9	4	11.4	0	0	14	27.5
third month	Anti-S	Positive	121	100	35	100	35	100	51	100
		Negative	0	0	0	0	0	0	0	0
	Anti-N (*p* = 0.003)	Positive	110	90.9	34	97.1	35	100	41	80.4
		Negative	11	9.1	1	2.9	0	0	10	19.6
Antibody Levels			Mean	SD	Mean	SD	Mean	SD	Mean	SD
sixth month	Anti-S (*p* < 0.001)		121.91	89.74	109.35	84.46	172.74	81.64	95.65	85.58
	Anti-N (*p* < 0.001)		38.68	57.10	50.27	60.55	72.05	69.13	7.84	15.48
third month	Anti-S (*p* < 0.001)		136.52	89.80	105.81	82.12	208.59	64.78	108.15	82.44
	Anti-N (*p* < 0.001)		60.93	71.81	71.84	67.39	117.66	78.88	14.51	22.88

Units of U/mL were used for Anti-S IgG, and units of COI were anti-N IgG/IgM. Group 1: Participants who had been diagnosed with COVID-19 and who had not yet been vaccinated. Group 2: Participants who had been diagnosed with COVID-19 and who were subsequently vaccinated with CoronaVac. Group 3: Participants who had been vaccinated with two doses of CoronaVac in the Turkish Ministry of Health’s vaccination program (without prior diagnosis of COVID-19).

**Table 3:** Mean differences in antibody levels between groups.

Sixth Month Anti-S (121)		Reference Groups	Mean Difference	95% CI		*p*
Groups	Group 2	Group 1	63.39	23.77	103.02	0.002
	Group 2	Group 3	38.55	20.21	56.88	<0.001
	Group 1	Group 3	4.57	−7.82	16.95	0.080
Sixth month Anti-N (121)						
Groups	Group 2	Group 1	21.78	−9.22	52.78	0.165
	Group 2	Group 3	32.11	22.16	42.05	<0.001
	Group 1	Group 3	14.14	8.28	20.01	<0.001
Third month Anti-S (121)						
Groups	Group 2	Group 1	92.58	59.78	125.38	<0.001
	Group 2	Group 3	51.19	35.5	66.88	<0.001
	Group 1	Group 3	3.27	−7.5	14.03	0.059
Third month Anti-N (121)						
Groups	Group 2	Group 1	27.19	−4.17	58.56	0.088
	Group 2	Group 3	50.54	39.54	61.54	<0.001
	Group 1	Group 3	24.63	17.75	31.51	<0.001

Group 1: Participants who had been diagnosed with COVID-19 and who had not yet been vaccinated. Group 2: Participants who had been diagnosed with COVID-19 and who were vaccinated subsequently with CoronaVac.Group 3: Participants who have been vaccinated with two doses of CoronaVac in the Turkish Ministry of Health vaccination program (without prior diagnosis of COVID-19).

## Data Availability

The data presented in this study are available on reasonable request from the corresponding author.

## Abbreviations

Anti-N	Anti-Nucleocapsid IgG/IgM
Anti-S	Anti-Spike IgG
BAU	Binding antibody units
COI	Cut-off index
COVID-19	Coronavirus Disease 2019
HCWs	Healthcare workers.
NPs	Nasopharyngeal swab samples
OHSU	Occupational Health and Safety Unit
RBD	Receptor binding domain
RT-PCR	Reverse transcriptase-polymerase chain reaction
SPSS	Statistical package for social sciences
